# Eating Behaviour in the General Population: An Analysis of the Factor Structure of the German Version of the Three-Factor-Eating-Questionnaire (TFEQ) and Its Association with the Body Mass Index

**DOI:** 10.1371/journal.pone.0133977

**Published:** 2015-07-31

**Authors:** Antje Löffler, Tobias Luck, Francisca S. Then, Claudia Sikorski, Peter Kovacs, Yvonne Böttcher, Jana Breitfeld, Anke Tönjes, Annette Horstmann, Markus Löffler, Christoph Engel, Joachim Thiery, Arno Villringer, Michael Stumvoll, Steffi G. Riedel-Heller

**Affiliations:** 1 Institute of Social Medicine, Occupational Health and Public Health (ISAP), University of Leipzig, Leipzig, Germany; 2 LIFE-Leipzig Research Center for Civilization Diseases, University of Leipzig, Leipzig, Germany; 3 IFB AdiposityDiseases, Leipzig University Medical Center, Leipzig, Germany; 4 Division of Endocrinology and Nephrology, Medical Department, University of Leipzig, Leipzig, Germany; 5 Max Planck Institute for Human Cognitive and Brain Sciences, Leipzig, Germany; 6 Institute for Medical Informatics, Statistics and Epidemiology (IMISE), University of Leipzig, Leipzig, Germany; 7 Institute of Laboratory Medicine, Clinical Chemistry and Molecular Diagnostics, University of Leipzig, Leipzig, Germany; 8 Day Clinic of Cognitive Neurology, University of Leipzig, Leipzig, Germany; Charité-Universitätsmedizin Berlin, Campus Benjamin Franklin, GERMANY

## Abstract

The Three-Factor-Eating-Questionnaire (TFEQ) is an established instrument to assess eating behaviour. Analysis of the TFEQ-factor structure was based on selected, convenient and clinical samples so far. Aims of this study were (I) to analyse the factor structure of the German version of the TFEQ and (II)—based on the refined factor structure—to examine the association between eating behaviour and the body mass index (BMI) in a general population sample of 3,144 middle-aged and older participants (40–79 years) of the ongoing population based cohort study of the Leipzig Research Center for Civilization Diseases (LIFE Health Study). The factor structure was examined in a split-half analysis with both explorative and confirmatory factor analysis. Associations between TFEQ-scores and BMI values were tested with multiple regression analyses controlled for age, gender, and education. We found a three factor solution for the TFEQ with an ‘uncontrolled eating’, a ‘cognitive restraint’ and an ‘emotional eating’ domain including 29 of the original 51 TFEQ-items. Scores of the ‘uncontrolled eating domain’ showed the strongest correlation with BMI values (partial r = 0.26). Subjects with scores above the median in both ‘uncontrolled eating’ and ‘emotional eating’ showed the highest BMI values (mean = 29.41 kg/m^²^), subjects with scores below the median in all three domains showed the lowest BMI values (mean = 25.68 kg/m^²^; F = 72.074, p<0.001). Our findings suggest that the TFEQ is suitable to identify subjects with specific patterns of eating behaviour that are associated with higher BMI values. Such information may help health care professionals to develop and implement more tailored interventions for overweight and obese individuals.

## Introduction

Overweight and obesity are major public health problems worldwide. Regarding Germany, for example, 53.0% of the women and 67.1% of the men are overweight of whom 23.3% and 23.9% are obese [1]. Overweight and obesity are risk factors for a wide range of common non-communicable diseases such as coronary heart disease, hypertension, stroke, certain types of cancer, diabetes mellitus, gallbladder disease, dyslipidaemia, gout, and sleep apnoea [2]. The economic burden of obesity and its comorbidities is high: based on an obesity prevalence of 16 million patients costs are estimated to amount €20.26 billion/year alone in Germany [3].

Understanding the underlying mechanisms of becoming overweight and obese therefore is of utmost importance. To the best of the current knowledge, various interacting factors are involved in the development of overweight and obesity including genetic, metabolic, environmental, and socio-cultural aspects but also the individual eating behaviours [4].

Previous research of eating behaviour found different behavioural aspects which might cause weight problems. A common and well-established self-rating questionnaire for such eating behaviour aspects is the Three-Factor-Eating-Questionnaire (TFEQ) [5]. The 51-items-questionnaire measures three aspects of eating behaviour: (i) ‘cognitive restraint’ as the degree of cognitive control in daily food intake, (ii) ‘disinhibition’ as the loss of control in food intake, and (iii) ‘hunger’ as the susceptibility for internal or external hunger signs. The factor construction of the original TFEQ, however, was based on a small and selected convenient sample (members of a weight reduction program; n = 220). Further studies also conducted factor analyses of the TFEQ, but failed to replicate the original factor structure [6–10]. These studies, however, were also based on selected, convenient or clinical samples focusing primarily on younger subjects. Aim of our study therefore was to analyse the factor structure of the German version of the TFEQ in a non-clinical general population sample of middle aged and older adults (40–79 years). Based on the results of the factor analysis, we then examined the association between the results in the questionnaire and the body mass index (BMI) as an important defining measure of overweight and obesity.

## Methods

### Ethics statement

All participants provided written informed consent prior to their participation in the study. The study complies with the ethical standards of the Declaration of Helsinki and has been approved by the ethics committee of the University of Leipzig, Germany.

### Participants

**Fig 1:**
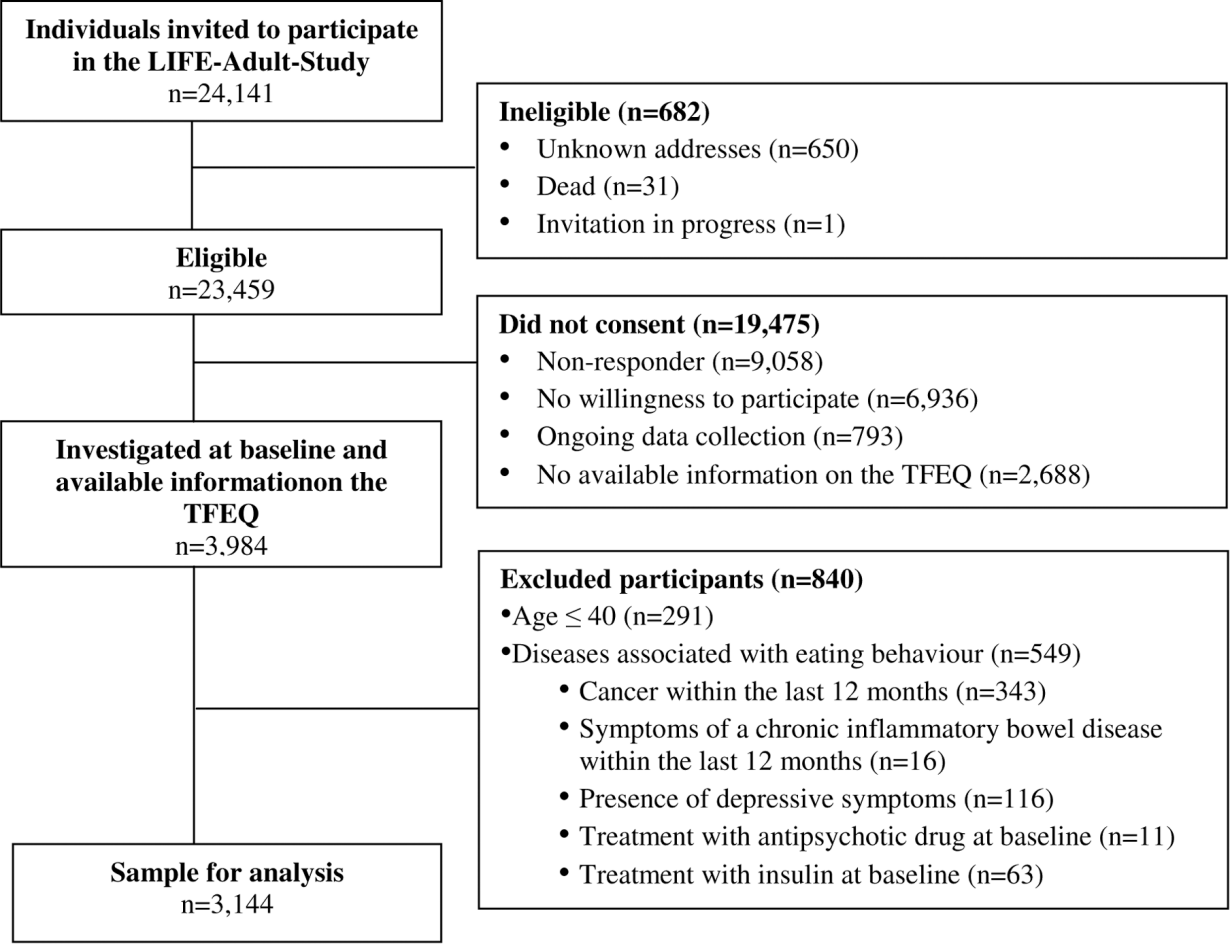
Sample attrition and sample for the Three-Factor-Eating-Questionnaire (TFEQ) factor analysis.

Data were derived from participants of the ongoing population based cohort study of the Leipzig Research Center for Civilization Diseases (LIFE) in Leipzig, Germany (LIFE Health Study). The LIFE Health study aims to examine causes for the development of important civilisation diseases such as obesity, diabetes, cardiovascular diseases, dementia, and allergies. Participants were identified by systematic random sampling from age-ordered lists provided by the Leipzig registry office. A detailed overview of the sample attrition of the study is shown in Fig 1. We excluded datasets from those participants who were afflicted by diseases and symptoms that might have caused significant changes in eating behaviour including cancer, symptoms of an inflammatory bowel disease, intake of antipsychotic drugs, insulin treatment, and depressive symptoms. We identified depressive symptoms using the German version of the 20-item Center of Epidemiologic Studies Depression Scale (CES-D; cut-off ≥ 23 points) [11–13]. Overall, the present analyses were based on a final sample of 3,144 subjects aged 40 to 79 years.

### Data collection and assessment procedure

Participants underwent a comprehensive assessment program including a variety of clinical examinations, clinical interviews, and standardized questionnaires (including those on socio-demographics and eating behaviour; see below). All examinations were conducted by trained study personnel at the LIFE research centre located on the premises of the University Hospital of Leipzig.

### Assessments

#### German version of the Three-Factor-Eating-Questionnaire

Eating behaviour was assessed with the “Fragebogen zum Essverhalten” (FEV), the German version of the Three-Factor-Eating-Questionnaire [5,10]. The original version of the TFEQ contains 51 items which cover three domains of the human eating behaviour: ‘cognitive restraint’, ‘disinhibition’ and ‘hunger’. The 21-item domain ‘cognitive restraint’ is characterized by a permanent cognitive control of food intake and cognitive override of physiological signs of hunger in order to maintain or to reduce body weight. The ‘disinhibition’ domain with 16 items describes a loss of control in food intake by various external or internal circumstances such as eating in society or in emotional mood, and the ‘hunger’ domain includes 14 items which describes the exceeding sense of internal hunger signs [5,10].

All TFEQ items are coded with either 0 or 1 point leading to maximum sum scores of 21 points for the domain of ‘cognitive restraint’, 16 points for ‘disinhibition’ and 14 points for ‘hunger’. Higher scores indicate stronger characteristic values in the domains.

Overall, the German version of the TFEQ was found to be a valid and reliable instrument for assessing eating behaviour, so far. Validation study showed satisfying results for all three domains with Cronbach’s alpha 0.74 to 0.87. However, the authors claimed that factor structure of the TFEQ may need improvement (e.g. further item selection) [5,10]. Also, as mentioned above (see introduction), results of the validation study were based on selected, convenient and clinical samples focusing primarily on younger subjects and not on population-based samples.

#### Anthropometric measurement

Anthropometric measurement was conducted by trained study personal. Body weight was measured with scale SECA 701, height was measured with height rod SECA 220 (SECA Gmbh & Co. KG). Body mass index (BMI) was calculated as the weight in kilograms divided by the square height in meters (kg/m^**2**^).

### Statistical Analyses

The statistical analyses were performed using Predictive Analytic Software (PASW) Version 20.0 (IBM Corp., Armonk, NY, USA). All analyses employed an alpha level for statistical significance of 0.05 (two-tailed).

To examine the factor structure of the German version of the TFEQ, we conducted a split-half factor analysis. To divide the sample, we used the SPSS random case selection procedure. On the random first split-half sample, we applied Principal Axis Factor analysis (PAF) with varimax rotation as an Explorative Factor Analysis (EFA). The PAF identifies a number of factors underlying the items of the TFEQ. Determination of the number of factors that fit best to the items was based on the following steps: (i) applying the criterion of Kayser-Mayer-Olkin that determines the number of factors which explain more variance as an item itself (eigenvalues greater than 1), (ii) examination of the scree plot: The scree plot shows the eigenvalues on the y-axis and the number of factors on the x-axis and displays a corresponding downward curve. The point where the slope of the curve is clearly levelling off indicates the number of factors that should be generated by the analysis, and (iii), conduction of a second PAF: In this PAF, we determined a priori the number of factors according to the results of the scree test and defined that items attaining a factor loading of ≥0.40 will be included in the factor structure model to ensure that these items load on existing underlying factors [14].

On the random second split-half sample, we conducted Confirmatory Factor Analysis (CFA) to test whether the identified factor structure of the PAF fits to the items of the TFEQ. We used common indices to examine whether the model was acceptable to the obtained data: the absolute fit indices Goodness of Fit Index (GFI) and Standardized Root Mean Square Residual (SRMR), and the Comparative Fit Index (CFI) and Root Mean Square Error of Approximation (RMSEA) as comparative fit indices to assess the model fit. The GFI measures the fit between the observed model and the covariance matrix. A GFI value >0.9 indicates a good model fit. The SRMR measures the difference between the observed model correlations and the predicated model correlation. A SRMR less than 0.08 is considered as a good model fit [15]. In contrast to the absolute model fit indices, RMSEA and CFI compare the fit between the target model and an independence model, wherein all variables are uncorrelated. The CFI measures the discrepancy of the approximation of the models as a ‘goodness of fit model index’. Higher CFI values indicate better obtained model fit (values above 0.90 indicate a good model fit) [15]. The RMSEA measures the discrepancy of the approximation of the models as a ‘badness of fit model index’. Here, lower RMSEA values indicate a better obtained model fit. Specifically, an RMSEA of 0.01 indicates an excellent fit, an RMSEA of 0.05 and lower indicates a good fit and a fit of 0.08 and higher indicates a poor fit of the model to the obtained data [15]. Additionally to the model fit, we also examined the internal consistency of the refined factors calculating the Cronbach’s alpha coefficient [14].

Based on the results of the factor analyses, in a last step, we analysed the association between the identified factors of eating behaviour and BMI values. For that, we conducted stepwise backward multiple regression analysis. In the regression model, we included the sum scores of the revised TFEQ factors, age, gender, and education as the independent variables and the BMI value as the dependent variable. Linear Z-transformation of the metric scales was obtained to ensure comparability of the included variables. The partial regression squared coefficient was used as a measure of effect size, which indicates the proportion of variance of the BMI values as the dependent variable when the included independent variables held constant. The r-squared value was used to provide an extent of BMI’s variability which is explained by the variables included into the model.

Educational level of the participants was classified as low, medium or high according to the CASMIN classification system [16].

## Results

### Sample description

The studied 3,144 participants (52.1 male, 47.9% female) were at a mean age of 54.97 years (SD ±9.70, range 40–79 years). The mean BMI was 27.19 kg/m² (SD±4.71 kg/m², range 15.72–55.36 kg/m²). Education was low in 2.9%, middle in 56.4% and high in 40.7% of the participants.

### Analysis of the factor structure of the TFEQ

Based on the data of one half of the sample (n = 1,574), we conducted the explorative factor analysis (Principal Axis Factor analysis, PAF) of the TFEQ. PAF identified 12 factors (Kayser-Mayer-Olkin-Eigenvalues >1.0) that explained 49% of the total variance (see Table 1).

**Table 1:** Kayser-Mayer-Olkin-Eigenvalues of the TFEQ-items in the PAF.

Kayser-Meyer-Olkin Eigenvalues			
Factor	Initial Eigenvalues	% of variance	Cumulative %
1	6.342	12.44	12.44
2	5.454	10.69	23.13
3	1.664	3.26	26.39
4	1.592	3.12	29.51
5	1.478	2.90	32.41
6	1.447	2.84	35.25
7	1.270	2.49	37.74
8	1.224	2.40	40.14
9	1.174	2.30	42.44
10	1.096	2.19	44.59
11	1.076	2.11	46.70
12	1.032	2.02	48.72

Examination of the scree plot then indicated a solution with the first three of the identified factors. Conducting the second PAF with factor loadings of ≥0.40 supported this three-factor-solution. According to the original TFEQ, we also identified a ‘cognitive restraint’ factor (first factor). Items of the original TFEQ ‘hunger’ and ‘disinhibition’ domains constituted the second factor which we labelled ‘uncontrolled eating’. And finally, the identified third factor included items of the origin ‘disinhibition’ domain that describe disinhibition in eating caused by emotional triggers like feeling anxious or depressed. We therefore labelled this factor ‘emotional eating’.

Based on the data of the other half of the sample, we then conducted the confirmatory factor analysis to test whether the identified factor structure of the PAF fits to the items of the TFEQ. Indices showed acceptable model fit with GFI = 0.928, SRMR = 0.0518, CFI = 0.885 and RMSEA = 0.046.

Overall, the ‘cognitive restraint’ domain contained 15 items, the identified ‘uncontrolled eating’ domain contained 11 items and the ‘emotional eating’ domain contained 3 items. Table 2 shows these items and their factor loadings in the EFA and CFA according to the numbering and factor attribution of the German version of the TFEQ [5,10].

**Table 2:** Factor loading of the remained items in EFA and CFA.

original TFEQ*	factor loadings					
	restrained eating		uncontrolled eating		emotional eating	
	EFA	CFA	EFA	CFA	EFA	CFA
RS58	0.654	0.628				
RS36	0.625	0.658				
RS53	0.589	0.576				
RS41	0.573	0.588				
RS14	0.552	0.509				
RS49	0.528	0.120				
RS46	0.517	0.587				
RS26	0.474	0.476				
RS38	0.472	0.501				
RS48	0.461	0.458				
RS57	0.461	0.457				
RS43	0.432	0.498				
RS22	0.415	0.434				
RS40	0.403	0.460				
RS56	0.402	0.393				
HU34			0.587	0.582		
HU13			0.586	0.638		
DS09			0.532	0.566		
DS10			0.520	0.509		
HU27			0.482	0.517		
DS15			0.466	0.481		
DS23			0.454	0.523		
DS21			0.447	0.594		
HU32			0.442	0.511		
HU16			0.434	0.486		
HU30			0.427	0.458		
DS28					0.685	0.840
DS17					0.589	0.657
DS35					0.586	0.729

*DS = ‘disinhibition’ domain, HU = ‘hunger’ domain, RS = ‘cognitive restraint’ domain. Item numbering correspondents to the origin German version of the TFEQ (includes item numbers ‘9 to 59’) [10].

To test the internal consistency of the refined factors, we calculated Cronbach’s alpha reliability coefficients. The factor ‘restrained eating’ showed a Cronbach’s alpha of 0.840, the factor ‘uncontrolled eating of 0.802, and the factor ‘emotional eating’ of 0.780.

### Analysis of the association between the scores in the TFEQ-domains and the BMI

Stepwise backward multiple regression analysis revealed that ‘uncontrolled eating’, emotional eating’ and ‘restrained eating’ were significantly associated with the BMI along with age, gender, and education (see Table 3). The regression weights (B-weight) and partial correlation coefficient (partial r²) of ‘emotional eating’ and ‘restrained eating’ indicated a small but positive association with the BMI values (B-weight = 0.38, r² = 0.08; B-weight = 0.45, r² = 0.26), the regression weight of ‘uncontrolled eating’ indicated the strongest positive correlation with the BMI values (B-weight = 1.34, partial r = 0.26).

**Table 3:** Multiple regression analysis for the association between TFEQ-eating behaviour factors and BMI, controlled for age, gender, and education (r² = 0.146).

	B-weight	SE	t-values	p-values	95.0% CI lower bound	95.0% CI upper bound	partial r²
intercept	30.78	0.45	68.58	<0.001	29.90	31.66	
restrained *	0.38	0.08	4.48	<0.001	0.21	0.54	0.08
uncontrolled*	1.34	0.09	15.04	<0.001	1.16	1.51	0.26
emotional *	0.45	0.09	4.96	<0.001	0.27	0.63	0.09
education	-0.75	0.15	-5.15	<0.001	-1.04	-0.47	-0.09
age*	0.80	0.08	9.86	<0.001	0.64	0.96	0.17
sex	-1.22	0.17	-7.19	<0.001	-1.55	-0.89	-0.13

*z-values, SE = standard error, CI = confidence interval.

The examined model was found to have an R-squared value of about 0.146 indicating that approximately 15% of the variability of the BMI was explained by the variables included into the model.

In an additional step, we examined the BMI values in association with the participants’ eating behaviour in all three TFEQ-domains. Based on the median split of the results in each of the revised TFEQ-domain, we therefore divided the sample in ‘cognitively restrained’ (n = 1,483) and ‘cognitively unrestrained eaters’ (n = 1,661), ‘uncontrolled’ (n = 1,450) and ‘controlled’ eaters (n = 1,694), and ‘emotional’ (n = 683) and ‘unemotional eaters’ (n = 2,461). We found that subjects who were both ‘uncontrolled’ and ‘emotional eaters’ (but not restrained eaters) had the highest mean BMI (29.55 kg/m²/SD = 5.93 kg/m²; n = 215), followed by subjects who were ‘uncontrolled’, ‘emotional’ and ‘restrained eaters’ (mean/SD BMI = 28.98/5.10 kg/m²; n = 316), subjects who were ‘uncontrolled’ and ‘restrained eaters’ (mean/SD BMI = 28.00/4.59 kg/m²; n = 381), and subjects who were ‘emotional’ and ‘restrained eaters’ (mean SD/BMI = 26.59/4.70 kg/m²; n = 96). The lowest mean BMI was found in those subjects who scored low in all three TFEQ-domains, i.e. who were neither ‘uncontrolled’, nor ‘restrained’, nor ‘emotional eaters’ (mean/SD BMI = 25.64/4.00 kg/m²; n = 856). The differences in BMI between these eating behaviour groups were significant (univariate analysis of variance: F = 63.94, p<0.001). A gender-specific examination of the BMI values in the different eating behaviour groups showed comparable distributions for both genders (data not shown).

## Discussion

In this study, we sought to analyse the factor structure of the German version of the Three-Factor Eating Questionnaire (TFEQ) based on a large population-based sample of 3,144 middle-aged and older individuals. We found a three-factor-solution that contained one of the three original factors of the TFEQ: ‘cognitive restraint’. A second factor we labelled ‘uncontrolled eating’ due to the tendency to lose control over food intake as it contained items of the original hunger and disinhibition factors. Instead of the third original factor of the TFEQ, ‘hunger’, our factor solution, however, identified another factor that we labelled ‘emotional eating’ as it contained items of disinhibition in eating caused by emotional triggers like feeling anxious or depressed. Model fit indices indicated an acceptable model fit. Reliability for these three identified factors was good and showed improved Cronbach’s alpha values when compared to the original German version of the TFEQ [5,10].

Our findings corroborate findings from several previous studies that also found a comparable three-factor-solution for the TFEQ, i.e. a solution including one domain that describes a cognitive control of food intake in order to lose weight or to maintain body weight, one domain that describes an increased food intake triggered by internal or external signs, and one domain that describes a behaviour of emotional eating [6–8,10]. To the best of our knowledge, our study, however, was the first providing TFEQ-factor analysis results based on a large general population sample.

The second aim of our study was to examine the association between the scores in the identified three TFEQ-factors and the BMI values of the participants.

The results of the multiple regression analysis suggested that higher scores in ‘uncontrolled eating’, emotional eating’ and ‘restrained eating’ are significantly associated with higher BMI values. The strongest positive association was found for ‘uncontrolled eating’ and BMI. This association corroborates results of others as a review of French et al. (2012) reported a consistent association between ‘eating disinhibition’—which is synonymous for ‘uncontrolled eating’- and BMI or weight gain in ten out of eleven cross-sectional studies and in seven out of nine prospective studies [17].

In contrast to our result, some previous studies reported significant negative associations between ‘cognitive restraint’ and BMI. Johnson et al., for example, found that higher cognitive restraint scores were associated with lower BMI values in overweight subjects but with higher BMI values in normal weight subjects [18]. The authors accordingly suggested that in the group of overweight subjects high cognitive restraint might temper food intake, and thus, lead to a lower BMI, whereas high cognitive restraint in normal weight subjects might increase the risk of overeating tendencies, and thus, lead to a higher BMI [18]. Westenhöfer even differentiated two types of ‘cognitive restraint’: a flexible and a rigid one. While a flexible cognitive control might lead to a successful control of body weight, a rigid one seems to be associated with a less effective weight control, and thus, higher BMI values [19–21]. The association between ‘cognitive restraint’ and BMI, however, might be moreover complex, as there might also be an interaction with the ‘disinhibition’ domain of eating behavior. In particular, recent findings have shown an inverted u-shaped relationship between restrained eating and BMI, which was further moderated by the level of disinhibited eating [22]. In additional analyses, we also found an inverted u-shape relationship between the restrained score and BMI (data not shown), indicating higher BMI values when subjects had a medium cognitive restraint score and lower BMI values when subjects scored high or low on the cognitive restraint factor. Further studies might be required to analyse such specific aspects of and interactions in the association between ‘cognitive restraint’ and BMI.

Regarding our third identified factor of eating behaviour, ‘emotional eating’, we found a weak positive association between higher scores in this factor and the BMI values. As stated above, high ‘emotional eating’ describes an increased food intake as a strategy to regulate adverse emotions like feeling lonely, anxious, or depressed–i.e. subjects with such a strategy eat more than usual in a dysphoric mood. Accordingly, a study from Geliebter et al. examined eating during emotional states and situations using an ‘appetite questionnaire’. This questionnaire covers positive and negative emotional states and their effect on food intake. The authors found that high ‘emotional eaters’, who eat when having negative feelings like feeling anxious or depressed, were more often obese than subjects using other mood regulation strategies [23].

Our findings also corroborate results of Koenders et al. who analysed the association between eating behaviour and weight in a two-year follow up study on office-working employees. Using the Dutch Eating Behaviour Questionnaire (DBEQ), which also measures three domains of eating behavior (‘emotional eating’, ‘uncontrolled eating’ and ‘restrained eating’), the authors found a significant association between high scores in the emotional eating domain and weight gain. [24]. And finally, in a study by Horstmann et al., in women there was found an association between higher emotional eating values and a common genetic variation which is associated with obesity [25].

In the last step of our study, we examined the association between different patterns of eating behaviour and the BMI values of the participants. When combining the characteristics of the revised TFEQ-factors, we found that subjects who scored low in all three identified eating behaviour factors (scores lower than the median in the factors) had the lowest BMI values whereas subjects who were both ‘uncontrolled’ and ‘emotional eaters’ (scores higher than the median in the two factors, but not in the ‘cognitive restraint’ factor) had the highest BMI values. As subjects with this latter pattern of eating behaviour may have a specifically increased risk of overweight and obesity, they may thus constitute a group of particular clinical interest.

Our study is not without limitations. First, even though participants were randomly selected from age-ordered lists provided by the Leipzig registry office, the representativeness of our sample may be limited because of the study’s relatively low response rate. Second, information on eating behaviour was only self-reported and may thus introduce some bias. And finally, our findings are based on a sample of middle-aged and older subjects. Generalisation to younger subjects therefore has to be made with caution.

Irrespective of these limitations, the present study on a large population-based sample showed that the TFEQ is suitable to assess different dimensions of eating behaviour not only in clinical samples but also in the middle-aged and older general population. Particularly the focus on the middle-aged and older population is a strength of our study as these age groups where underrepresented in previous analyses. Importantly, we also found that the different dimensions of eating behaviour can be associated with different BMI values: a pattern of high uncontrolled and high emotional eating was associated with a particularly high BMI, whereas a pattern of low scores in all three dimensions ‘cognitive restraint’, ‘uncontrolled’, and ‘emotional eating’ was associated with the lowest mean BMI values. Information on such low and high risk eating behaviour groups may thus help health care professionals to develop and implement more tailored interventions for overweight and obese individuals.

## References

[1] Mensink G, Schienkiewitz A, Haftenberger M, Lampert T, Ziese T, Scheidt-Nave C (2013) Übergewicht und Adipositas in Deutschland. Bundesgesundheitsbl. 56 (5–6): 786–794. Accessed 9 July 2014. 10.1007/s00103-012-1656-3 23703499

[2] WHO (2000) Obesity: preventing and managing the global epidemic. Report of a WHO consultation. World Health Organ Tech Rep Ser 894: i–xii, 1–253. 11234459

[3] Effertz T (2013) Kosten von Adipositas in Deutschland. Available: http://www.wiso.uni-hamburg.de. Accessed 30 September 2014.

[4] Renner B, Sproesser G, Strohbach S, Schupp HT (2012) Why we eat what we eat. The Eating Motivation Survey (TEMS). Appetite 59 (1): 117–128. Available: http://www.sciencedirect.com/science/article/pii/S0195666312001286. 10.1016/j.appet.2012.04.004 22521515

[5] Stunkard AJ, Messick S (1985) The three-factor eating questionnaire to measure dietary restraint, disinhibition and hunger. Journal of Psychosomatic Research 29 (1): 71–83. Available: http://www.sciencedirect.com/science/article/pii/0022399985900108. 3981480 10.1016/0022-3999(85)90010-8

[6] Ganley RM (1988) Emotional eating and how it relates to dietary restraint, disinhibition, and perceived hunger. Int. J. Eat. Disord. 7 (5): 635–647. Available: 10.1002/1098-108X(198809)7:5<635::AID-EAT2260070507>3.0.CO;2-K.

[7] Hyland ME, Irvine SH, Thacker C, Dann PL, Dennis I (1989) Psychometric analysis of the Stunkard-Messick Eating Questionnaire (SMEQ) and Comparison with the dutch Eating Behavior Questionnaire (DEBQ). Current Psychology 8 (3): 228–233. Available: 10.1007/BF02686751.

[8] Karlsson J, Persson L-O, Sjoerstroem L, Sullivan M (2000) Psychometric properties and factor structure of the Three-Factor Eating Questionnaire (TFEQ) in obese men and women. Results from the Swedish Obese Subjects (SOS) study. Int J Obes 2000 (24): 1715–1725. Accessed 9 July 2014. 10.1038/sj.ijo.0801442 11126230

[9] Mazzeo SE, Aggen SH, Anderson C, Tozzi F, Bulik CM (2003) Investigating the structure of the eating inventory (three-factor eating questionnaire): A confirmatory approach. Int. J. Eat. Disord. 34 (2): 255–264. Available: 10.1002/eat.10180. 12898563

[10] Pudel V, Westenhöfer J (1989) Fragebogen zum Eßverhalten (FEV); Handanweisung. Göttingen: Verl. für Psychologie Hogrefe. 38 p.

[11] Hautzinger M (2012) Allgemeine Depressionsskala. ADS. Göttingen u.a: Hogrefe. Manual (60 S.); 20 Fragebogen ADS-L; p.

[12] Radloff LS (1977) The CES-D-Scale: A-Self-report-Depression Scale for Research in the General Population. Applied Psychological Measurement (1): 385–401. Accessed 1 October 2014.

[13] Stein J, Luppa M, Mahnke J, Weyerer S, Schomerus G, Riedel-Heller SG (2014) Depressionsscreening am Telefon mittels der Allgemeinen Depressionsskala (ADS). Psychiat Prax 41 (03): 135–141. 10.1055/s-0033-1343176 23670784

[14] Cronbach L (1951) Coefficient alpha and the internal structure of tests. Psychometrika 16 (3): 297–334. Available: 10.1007/BF02310555.

[15] Hu L, Bentler PM (1999) Cutoff criteria for fit indexes in covariance structure analysis: Conventional criteria versus new alternatives. Structural Equation Modeling: A Multidisciplinary Journal 6 (1): 1–55. Available: 10.1080/10705519909540118.

[16] Brauns H, Steinmann S (1999) Educational reform in France, West-Germany and the United Kingdom: Updating the CASMIN educational classification. ZUMA-Nachrichten 44: 7–44.

[17] French SA, Epstein LH, Jeffery RW, Blundell JE, Wardle J (2012) Eating behavior dimensions. Associations with energy intake and body weight. A review. Appetite 59 (2): 541–549. 10.1016/j.appet.2012.07.001 22796186 PMC3454469

[18] Johnson F, Pratt M, Wardle J (2012) Dietary restraint and self-regulation in eating behavior. Int J Obes (Lond) 36 (5): 665–674. 21829162 10.1038/ijo.2011.156

[19] Westenhoefer J, Stunkard AJ, Pudel V (1999) Validation of the flexible and rigid control dimensions of dietary restraint. Int J Eat Disord 26 (1): 53–64. 10349584 10.1002/(sici)1098-108x(199907)26:1<53::aid-eat7>3.0.co;2-n

[20] Stewart TM, Williamson DA, White MA (2002) Rigid vs. flexible dieting: association with eating disorder symptoms in nonobese women. Appetite 38 (1): 39–44. Available: http://www.sciencedirect.com/science/article/pii/S0195666301904453. 11883916 10.1006/appe.2001.0445

[21] Timko CA, Perone J (2005) Rigid and flexible control of eating behavior in a college population. Eat Behav 6 (2): 119–125. 15598598 10.1016/j.eatbeh.2004.09.002

[22] Dietrich A, Federbusch MG, Grellmann C, Villringer A, Horstmann A (2014) Body weight status, eating behavior, sensitivity to reward/punishment, and gender: relationships and interdependencies. Frontiers in Psychology 5 (1073). 10.3389/fpsyg.2014.01073 PMC4202791 25368586

[23] Geliebter A, Aversa A (2003) Emotional eating in overweight, normal weight, and underweight individuals. Eat Behav 3 (4): 341–347. 15000995 10.1016/s1471-0153(02)00100-9

[24] Koenders PG, van Strien T (2011) Emotional eating, rather than lifestyle behavior, drives weight gain in a prospective study in 1562 employees. J Occup Environ Med 53 (11): 1287–1293. 10.1097/JOM.0b013e31823078a2 22027541

[25] Horstmann A, Kovacs P, Kabisch S, Boettcher Y, Schloegl H, Tönjes A et al (2013) Common Genetic Variation near MC4R Has a Sex-Specific Impact on Human Brain Structure and Eating Behavior. PLoS ONE 8 (9): e74362 EP. Available: http://dx.doi.org/10.1371%2Fjournal.pone.0074362. 10.1371/journal.pone.0074362 24066140 PMC3774636

